# Developing a screening test for toxicity studies of prenatal development with the use of *Hydra attenuata* and embryos of zebrafish

**DOI:** 10.1016/j.toxrep.2021.09.006

**Published:** 2021-10-01

**Authors:** Robert Sornat, Joanna Kalka, Justyna Faron, Marta Napora-Rutkowska, Daniel Krakowian, Agnieszka Drzewiecka

**Affiliations:** a Łukasiewicz Research Network – Institute of Industrial Organic Chemistry, Branch Pszczyna, Doświadczalna 27, 43-200, Pszczyna, Poland; b Silesian University of Technology, The Faculty of Energy and Environmental Engineering, Konarskiego 18, 44-100, Gliwice, Poland; c Veterinary Clinic LUX-VET, Słoneczna 118, 43-384, Jaworze, Poland

**Keywords:** *Hydra attenuate*, Zebrafish embryos, Alternative methods

## Abstract

11 active substances used in pesticides were selected. Substances were divided into three groups depending the effect on embryos or fetuses of laboratory animals: 1 – damaging effect on embryos or fetuses (embryotoxic, fetotoxic or teratogenic), 2 – damaging effect on embryos or fetuses, but only at dose toxic for mother (maternal toxicity), 3 – no damaging effect.

Changes for hydra in acute toxicity tests and recovery tests were assessed on an change scale from 0 to 10. The index of the effect on development (TI) for hydras was calculated for every compound. Changes in zebrafish embryos were assessed using a descriptive method. Pearson correlation coefficient showed the correlation between the concentration and the toxic effect in the zebrafish embryos for the substances of the first group.

The study showed that substances having a strong damaging effect on fetuses cause changes that are apparent and easy to evaluate both in hydras and zebrafish embryos. A scoring system was introduced to evaluate the changes of hydras and zebrafish embryos. The point system of evaluation of changes allows quick classification of a substance as potentially embryotoxic, fetotoxic or teratogenic. It allows developing a cheap and fast method alternative to prenatal developmental toxicity studies, a screening method that enables substances of great teratogenic potential to be excluded from studies on laboratory animals.

## Highlights

• A simple alternative method may replace the laboratory animals in teratogenic studies.
• A scoring system evaluates the changes of H*ydra attenuata* and zebrafish embryos.
• A potentially teratogenic substance can be easily classified.

## Graphical abstract

**Figure:**
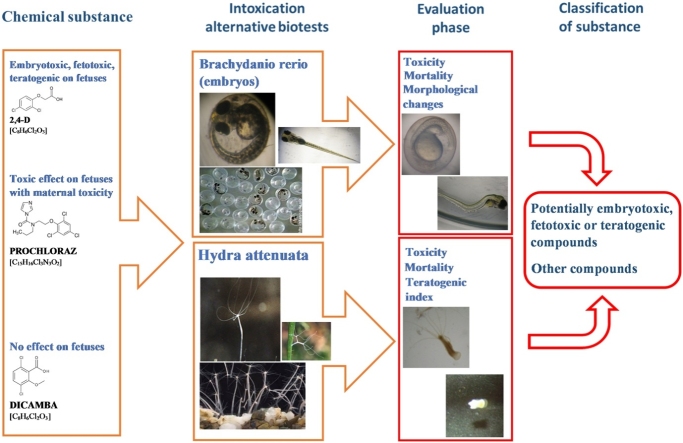

## 1 Introduction

A number of new chemical compounds are synthesized every year. Some of them are applicable and used in various fields of human activity. For safe usage of these compounds, it is necessary to understand their effect on human health and the environment. Toxicology and ecotoxicology studies, in some of which laboratory animals are used, allow to understand these effects.

Using laboratory animals in experiments had been ethically dubious for a long time. As a result, as early as in the 1950’s the 3R-rule (refining, reducing, replacing) concerning the use of animals was introduced. Economic factors are of equal importance. Studying the toxicity of prenatal development according to OECD Guideline 414 [1] requires using a hundred pregnant females and their fetuses, which frequently adds up to over a thousand animals. Such an experiment is estimated to last several months.

For a number of years the OECD has been successfully introducing alternative methods using cell cultures or tissue cultures, which has significantly contributed to decreasing the number of laboratory animals used in acute toxicity tests [2,3]. It is far more challenging, however, to come up with alternative methods to apply in experiments with a repeated dose, in which it is a system reaction of an organism that is assessed. It appears to be an acceptable solution to seek alternative methods in such studies using organisms that are not highly developed in terms of phylogenetics such as *Hydra attenuata* and *Danio rerio*.

*Hydra attenuata* is referred to in OECD Guidelines as a model organism for environmental studies and it is used as an indicator of the purity of water in a number of laboratories both in Poland [4,5] and across the world [6]. In the 1980’s *Hydra attenuata* began to be used in teratogenicity studies due to its ability to recover from cell homogenate [7,8]. Later this method was simplified by replacing cell homogenate with a gastric part of an adult specimen [9,10].

Since the 1980’s the zebrafish has been becoming a more and more important as a model organism in studies [11]. The easiness of breeding, a short developmental cycle and the fast growth of embryos as well as transparent chirons enable its development to be followed. This species is recommended in OECD Guidelines to be used in acute toxicity studies of embryos [12]. Possessing the equivalents of seventy percent of human genes causes the zebrafish to be useful in research into a number of human diseases [13]. Numerous papers on the zebrafish make it apparent that the zebrafish is useful in studies of the environment [[14], [15], [16], [17]], medicines [18,19], neurotoxicity [20,21], immunotoxicity [22] and cancer [23].

Pesticides are commonly used in the human activity and they can have a negative impact on human and animal health and the environment. Wide and multi-directional possible harmful effects of pesticides are the real problems in taking appropriate regulatory actions. An example is the controversy surrounding glyphosate, which is believed to cause endocrine disruption [24]. Apart from the direct harmful effects of pesticides, pesticide residues may pose an equally serious threat. Pesticide residues can produce long-term negative effects both in human and in environment ecosystems [25]. A big issue is recognition and understanding the effects of long-lasting exposure of various chemicals in small concentrations. This type of exposure can lead to detrimental consequences [24]. Both active substances of pesticides and the pesticides residues can be equally hazardous [25]. Therefore risk should be approached holistically, examining the impact of pesticides on both human health and the environment. Various model organisms such as *Channa punctata* [26] or *Danio rerio* [27] can be used in the research. The latter is particularly useful in research because, apart from adults, it is also possible to conduct research on its embryos [27].

The study was an attempt to check the suitability of hydra and zebrafish embryos for use in an alternative method to prenatal developmental toxicity studies on laboratory animals. Confirmation of suitability would give the opportunity to develop a fast screening method.

## 2 Methods and materials

### 2.1 Biological research system of the experiment: the animals

#### 2.1.1 Hydra

*Hydra attenuata* came from Blades Biological Limited Yew Tree House, East Sussex. They were farmed in round flat bottom beakers 20 cm in diameter and 8 cm high, in a standard medium consisting mostly of distilled water and calcium chloride, TES and EDTA buffers and 1 N NaOH as a stabilizer of the proper pH level [9,10]. During farming the temperature in the room ranged from 22 °C to 24 °C. Additionally, artificial light was provided to ensure a cycle of 12 h of light and 13 h of the dark. The hydras were fed freshly hatched larvae of *Artemia salina* (standard food produced by Ooo Biotrade, Barnaul, Russia and Ocean Star International, Inc. Snowville, USA).

#### 2.1.2 The test procedure for hydra

The method was to expose adult hydra individuals to increasing concentrations of the tested substance in acute toxicity tests as well as gastric parts in regeneration tests and to observe the changes that occur at regular time intervals: 24, 48, 72 and 96 h after the exposing started. The final results of tests after 96 h were used for evaluation and final classification of substances.

First, in tests on adult hydras, the concentration inducing lethality was found in preliminary tests (unpublished own research). Subsequently, acute toxicity tests on adult hydra and regeneration tests on gastric fragments were performed simultaneously. Both the acute toxicity test and the regeneration tests were performed in the same concentration range, which were chosen so that the highest concentration caused lethality and the lowest did not cause adverse effects. The results of tests after 96 h were used to calculate the LC50 value (the concentration for which 50 % individuals are dead) for acute toxicity of adults and the LC50 value (the concentration for which 50 % individuals are dead) for the gastric fragments of the regeneration test. These values enabled the calculation of the index of the effect on development (TI) for the LC50 values according to the formula [[7], [8], [9], [10]]:

$$\text{T}{\text{I}}_{\text{LC50}}=\frac{\text{LC5}0_{\text{acute}\;\text{toxicity}}}{\text{LC5}0_{\text{regeneration}\;\text{test}}}$$

In addition, the results of tests after 96 h were used to calculate the EC50 value (the concentration for which 50 % individuals are properly developed and not become dystrophic) for acute toxicity of adults and the EC50 value (the concentration for which 50 % individuals are fully regenerated) for the gastric fragments of the regeneration test. These values enabled the calculation of the index of the effect on development (TI) for the EC50 values according to the formula:

$$\text{T}{\text{I}}_{\text{EC50}}=\frac{\text{EC5}0_{\text{acute}\;\text{toxicity}}}{\text{EC5}0_{\text{regeneration}\;\text{test}}}$$

The tests were performed in sterile 12-well plates. Each test was performed on 2 plates, which allowed using a control group with standard medium and 7 increasing concentrations of a test item. Every well was filled with 4 ml of an appropriately concentrated test item in such a way that in the first three-wells column on the left there was the control group in the medium and increasing concentrations of the test item in every next one. In the acute toxicity tests in every well 3 hydra individuals and in the regeneration tests 3 gastric parts were randomly placed. Each test for a given series of concentrations was performed twice, that is why, 18 specimens and 18 gastric parts were exposed to every concentration. To minimize the uncontrolled dissolving of solutions in the test wells the hydras and gastric parts were first placed in beakers containing 4 ml of the target concentration and after a while they were transferred to the target wells of an appropriate column [9,10,28]. Gastric parts were obtained with the scalpel on a glass Petri dish.

#### 2.1.3 Acute toxicity and regeneration tests for hydra

The changes were classified according to a scale from 0 to 10, separately for acute toxicity and regeneration test [9,10]. 0 was assigned to a specimen that fell apart, 10 was assigned to a specimen that was properly developed. Changes from 0 to 5 are considered to be irreversible and therefore lethal.

For 18 specimens a mean was calculated for every concentration after 24, 48, 72 and 96 h of exposing.

The calculation of the mean value for 18 individuals in a given concentration enabled the comparison of the changes and reactions of treated hydra to the control group, however the results after 96 h were used for the index of the effect on development (TI). The results were obtained with the Kraber method (all animals among various doses are treated together and one dose should cause death of all animals and one dose should cause no effect).

#### 2.1.4 Zebrafish embryos

Zebrafish (*Danio rerio*) came from ‘Zebrafish Core Facility’ of International Institute of Molecular and Cell Biology in Warsaw. Fish in the age of 5–10 months were used for spawning.

The fish were kept in aquaria having a capacity of 30 l, in a standard medium consisting mostly of distilled water and calcium chloride, magnesium sulfate, sodium hydrogen carbonate and potassium chloride at 25−26 °C, in rooms with access to day light, males separated from females. In addition, artificial light was provided to ensure a regime of 12 h a day of the dark and 12 h a day of light. The fish were fed standard fish food - TetraMin. The embryos were obtained in a double-bottom aquarium preventing the adult fish from accessing the embryos lying on the bottom. The day before the experiment several fish were placed in the breeding aquarium, one female per two males. At night the aquarium was not provided with light access and the water temperature was raised to 26 °C – 27 °C. In the morning the breeding began and after approximately 2 h eggs were collected from the bottom of the aquarium.

#### 2.1.5 The test procedure for zebrafish embryos

The method was to expose the developing zebrafish embryos to an appropriate concentration of the test item and to observe the changes that occur at regular time intervals: 24, 48, 72 and 96 h after the exposing started. The final results of tests after 96 h were used for evaluation and final classification of substances. The Pearson correlation coefficient was used to evaluate the results for zebrafish embryos.

The concentrations were prepared in the same way as in case of the hydra tests. The only exception was that the plates after being filled with solutions were in an incubator for about 40−60 min in order to stabilize the temperature at 27 °C.

The embryos obtained during spawning were evaluated and selected using an inverted microscope. The embryos selected for testing were placed randomly into wells of 12-well plates analogously to hydras, and the plates were incubated at 27 °C.

Each test was performed simultaneously on control group (with standard medium) and maximum 7 increasing concentrations. Each test was performed 2 times for every series of concentrations, that is why 18 embryos were exposed to every concentration.

At first, every test item was tested using a wide range of concentrations (preliminary tests), which allowed determining the concentration that caused mortality or evident changes in the embryos (unpublished own research). Then, at least two further tests were performed in such a way that the lowest concentration caused the embryos to develop as properly as the control group and the highest concentration caused the mortality of the embryos or evident changes (main tests).

#### 2.1.6 The changes observed during the exposure of zebrafish embryos

Zebrafish embryos develop in a specific way in proper conditions, which allows determining the age of an embryo accurate to within a few hours. Within several hours after the experiment it is possible to determine the age of an embryo accurate to within a several minutes [29,30,31]. Using a microscope one can notice anatomic changes which are the evidence of the developmental disorder of embryos or larvae (after hatching).

The following changes could be observed:

- coagulation of an embryo - evidence of the death,
- lack of somites, evidence of the death of a fetus after clear body structures had developed,
- no separation of the tail from the yolk sac,
- no heartbeat,
- decrease in the frequency of the heartbeat (visible in the cardiac muscle as well as in the peripheral blood flow),
- weaker heart contractility (visible in the cardiac muscle as well as in the peripheral blood flow),
- pericardial edema,
- swelling of the yolk sac,
- swelling of the body,
- flexion of the spine,
- distortion of the tail,
- distortion of the eyes, fin and head,
- lack of eyes or fins.

When assessing the embryos or larvae, the absence or presence of changes at a given concentration was recorded.

#### 2.1.7 Preparing concentrations of the test items for hydra and zebrafish embryos

The tested items were dissolved or suspended in a medium appropriate for the hydra or zebrafish embryos. First, the highest concentration was prepared. Next, it was dissolved on and on and this way further concentrations were obtained. An ultrasonic washer and electromagnetic stirrer were used for stirring in order to prepare the first, highest concentration as well as to dissolve or suspend a test item in the medium. In case of two test items, bromfenvinfos and deltamethrin, which do not dissolve in water, it was necessary to use DMSO as an auxiliary substance to prepare proper concentrations. For these test items, 0.5 % DMSO solution in the medium was provided as a negative control.

#### 2.1.8 Checking the stability of the tested solutions/suspensions

In order to check whether the method of preparing the concentrations was the right one, the content of the tested items in the solutions/suspensions was determined. Furthermore, in order to determine the stability of the solutions/suspensions, the content of the tested items in the solutions/suspensions was determined a few days after they were prepared. Lack of developed and validated method of determining CCC, glyphosate, copper oxychloride and bromfenvinfos, made it impossible to determine their content, which meant that every day the tested concentrations were prepared and changed in the wells. The tests showed insufficient stability of deltamethrin, which made it necessary to daily prepare and change deltamethrin concentrations in the wells.

#### 2.1.9 Test items

11 test items were used in tests. They were divided into three groups: the first one with embryotoxic, fetotoxic and teratogenic effects on fetuses (strong teratogens), the second one with toxic effects on fetuses which was revealed in maternally toxic doses, and the third one with lack of fetotoxic and teratogenic effects. Information on the effects on fetuses, and the division into three presented groups are based on information from Toxnet, Pubchem, Material Safety Data Sheet (MSDS), US EPA Archive Document, FAO Specifications And Evaluations For Agricultural Pesticides. As for 2,4-D, Bromfenfinfos and Copper oxychloride data of own researches were available. Test items are presented in Table 1.

**Table 1:** Test items used in the tests and their effect on prenatal development.

Test item CAS number, Batch / Delivery number, apperance		
2,4-D CAS: 94-75-7	Batch number: A0290110. Beige, odorless powder. China.	Compound with embryotoxic, fetotoxic and teratogenic effects (strong teratogens)
Carbendazim CAS: 10605-21-7	Batch number: MKBT5464V. Powder white to light beige colour. Sigma - Aldrich.	
MCPA CAS: 94-74-6	Batch number: BCBQ8023V. Flaked solid, with white to light-beige colour. Sigma - Aldrich.	
Bromfenvinfos CAS: 33399-00-7	Batch number: 6B/17. Opalescent liquid. IPO Warsaw.	
Copper oxychloride CAS: 1332-65-6	Delivery number: OA12C14F. Manufacturer batch number: 931. Greenish-blue powder. Cu content 58.87%. China.	Toxic effects to fetuses are revealed in maternally toxic doses (maternal toxicity)
Glyphosate CAS: 1071-83-6	Batch number: 1C/16. Crystalline powder. IPO Warsaw.	
Prochloraz CAS: 67747-09-5	Batch number: 3C/14. Crystalline powder. IPO Warsaw. [32].	
Deltamethrin CAS: 52918-63-5	Batch number: 4C/14. Crystalline powder. IPO Warsaw.	
Linuron CAS: 330-55-2	Batch number: 4C/14. Crystalline powder. IPO Warsaw.	
Chlormequat (CCC) CAS: 999-81-5	Batch number: MKBX4778 V. White crystalline powder. Sigma - Aldrich.	Lack of fetotoxic and teratogenic effects
Dicamba CAS: 1918-00-9	Batch number: 4A/15. Crystalline powder. IPO Warsaw.	

## 3 Results and discussion

### 3.1 Test item concentrations used in the hydra tests

The concentrations of which effect on hydras was tested in acute toxicity tests and regeneration tests and that were used to calculate the index of the effect on development (TI) after 96 h are presented in Table 2.

**Table 2:** Concentrations of test items tested on hydra.

Compound	Concentrations (mg/L)							
2,4-D	0	7.32	9.59	12.56	16.64	21.56	28.24	37
Carbendazim	0	0.0003	0.0006	0.0012	0.0024	0.0049	0.098	0.0195
MCPA	0	5.179	6.733	8.753	11.379	14.79	19.23	25
Bromfenvinfos	0	0.132	0.198	0.296	0.444	0.667	1.000	0.5 % DMSO
Copper oxychloride	0	0.39	0.56	0.78	1.09	1.53	2.14	3.00
Glyphosate	0	8.8	13.2	19.8	29.6	44.4	66.7	100.0
Prochloraz	0	0.34	0.40	0.48	0.58	0.69	0.83	1.00
Deltamethrin	0	0.006	0.019	0.056	0.167	0.500	0.5 % DMSO	–
Linuron	0	20.0	24.1	28.9	34.7	41.6	50.0	60.0
Chlormequat (CCC)	0	673	875	1138	1479	1923	2500	–
Dicamba	0	15.6	21.8	30.6	42.9	60.0	–	–

The index of the effect on the development (TI) for LC50 value and EC50 value was calculated for every test item [6,7,9,10]. The results are presented in Table 3.

**Table 3:** Index of the effect on the development (TI) – results for *Hydra attenuata* after 96 h.

Compound	TI for LC50 LC50 – the concentration for which 50 % individuals are dead (mg/L)	TI for EC50 EC50 – the concentration for which 50 % individuals are fully regenerated for gastric parts or not become dystrophic for adults (mg/L)	
2,4-D	1.40 ± 0.01	1.70 ± 0.06	Compound with embryotoxic, fetotoxic and teratogenic effects (strong teratogens)
Carbendazim	2.04 ± 0.75	10.85 ± 6.85	
MCPA	1.16 ± 0.01	2.17 ± 0.30	
Bromfenvinfos	1.24 ± 0.02	1.80 ± 0.25	
Copper oxychloride	1.07 ± 0.05	1.29 ± 0.03	Toxic effects to fetuses are revealed in maternally toxic doses (maternal toxicity)
Glyphosate	1.24 ± 0.03	1.75 ± 0.23	
Prochloraz	1.27 ± 0.03	1.32 ± 0.16	
Deltamethrin	5.09 ± 0.90	1.97 ± 0.42	
Linuron	1.16 ± 0.02	1.14 ± 0.04	
Chlormequat (CCC)	1.14 ± 0.11	1.31 ± 0.13	Lack of fetotoxic and teratogenic effects
Dicamba	1.13 ± 0.02	1.65 ± 0.04	

### 3.2 Review of the *Hydra attenuata* tests

The higher index of the effect on the development (TI) a given test item has, the more toxic it is for developing specimens of hydra, which might result in causing teratogenic changes. It appears that the index (TI) calculated on the basis of EC50 is more appropriate since EC50 characterizes the development and growth of hydra specimens from the gastric stage to adulthood, which better reflects the essence of the process, unlike LC50, in which it is mortality rate that is a parameter.

The index of the effect on the development (TI) for carbendazim and MCPA calculated on the basis of LC50 or EC50 exceeds 2 as they are strong teratogens and for deltamethrin, whose developmental toxicity is connected to maternal toxicity. It ought to be pointed out the deltamethrin is the only test item whose index (TI) calculated on the basis of LC50 is higher than the index (TI) calculated on the basis of EC50. The index (TI) is equal or exceed 1.7 for 2,4-D and bromfenvinfos as strong teratogens, and glifosat, whose toxicity to fetuses is connected with higher toxic doses for mothers [33,34]. Dicamba, considered as harmless for fetuses, had a relatively high index (TI), that is 1.65. The index (TI) of test items which toxicity is connected with mother toxicity such as prochloraz, linuron, copper oxychloride and chlormequat (CCC) as a substance harmless for fetuses, hardly exceeded 1.

The index (TI) of all the tested strong teratogens (2,4-D, karbendazym, MCPA and bromfenvinfos) was higher than the index of the other test items, with an exception of deltamethrin. However, the indices do not allow determining a clear borderline between a strong teratogen and a substance having a negative, toxic effect on fetuses with maternal toxicity. The indices of some of these test items were hardly lower than the lowest index (TI) of a strong teratogen. It can be concluded that a high index (TI) clearly exceeding 2 is very likely to characterize a test item as potentially teratogenic. Indices slightly lower than 2 seem to characterize both strong teratogens and non-teratogenic substances or substances having no damaging impact of fetuses. The hydra study and the index (TI) calculated on its basis might be referred to as a kind of a forecast. However, the index (TI) itself cannot be used to classify a given test item as teratogenic and can be complementary to other studies, e.g. studies on zebrafish embryos.

### 3.3 Test item concentrations used in the zebrafish embryo tests

**Fig. 1:**
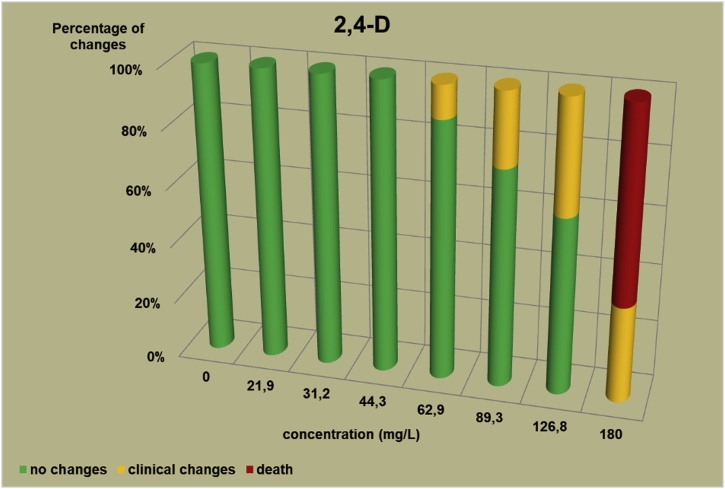
2,4-D – changes in zebrafish embryos after 96 h of exposure.

**Fig. 2:**
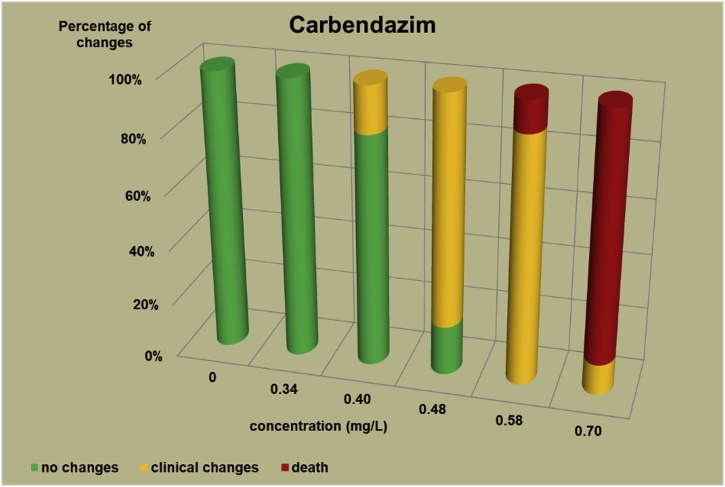
Carbendazim – changes in zebrafish embryos after 96 h of exposure.

**Fig. 3:**
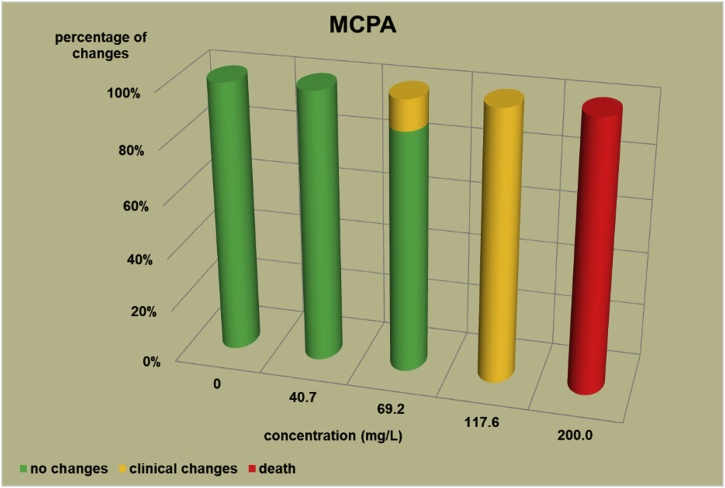
MCPA – changes in zebrafish embryos after 96 h of exposure.

**Fig. 4:**
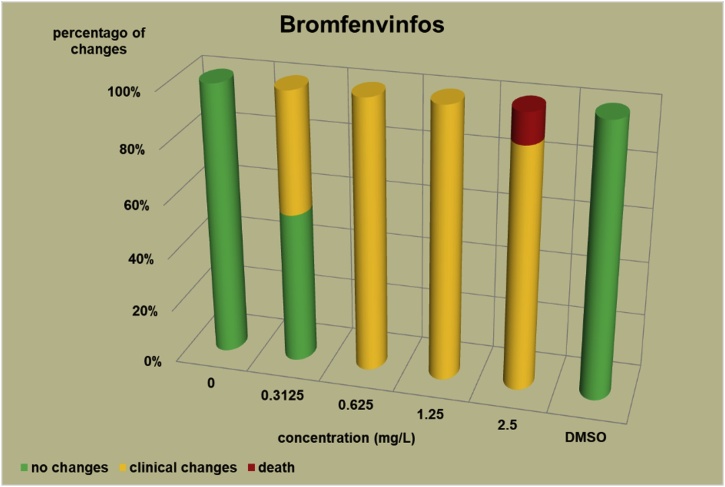
Bromfenvinfos – changes in zebrafish embryos after 96 h of exposure.

**Fig. 5:**
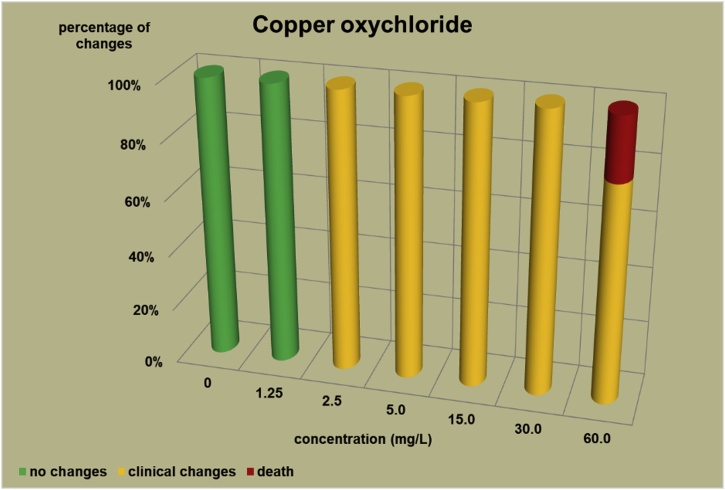
Copper oxychloride – changes in zebrafish embryos after 96 h of exposure.

**Fig. 6:**
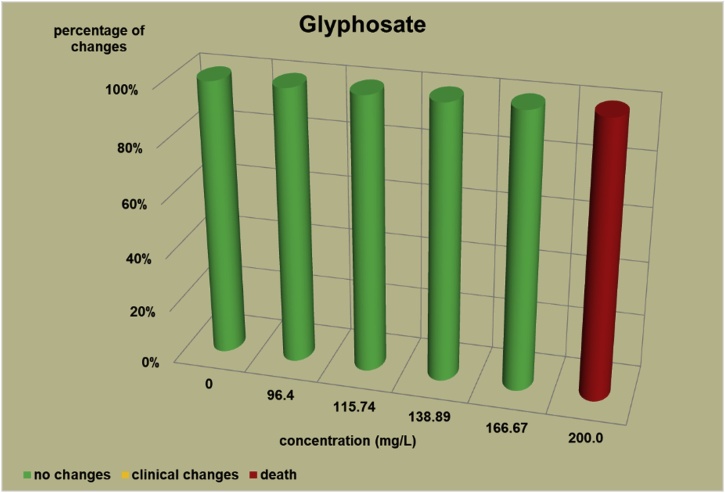
Glyphosate – changes in zebrafish embryos after 96 h of exposure.

**Fig. 7:**
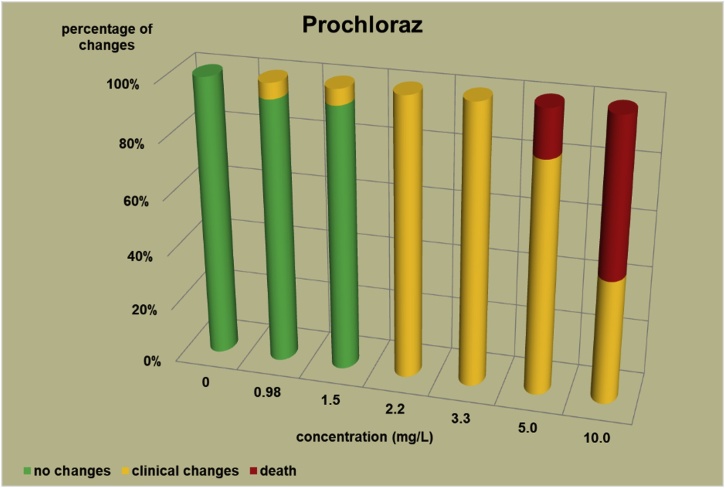
Prochloraz – changes in zebrafish embryos after 96 h of exposure.

**Fig. 8:**
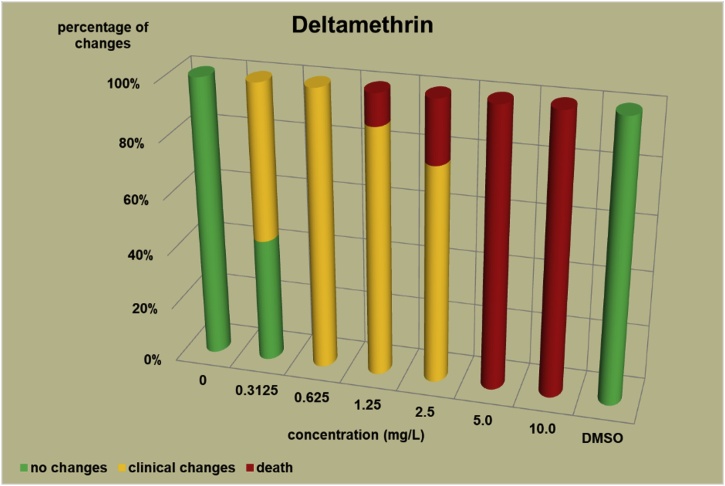
Deltamethrin – changes in zebrafish embryos after 96 h of exposure.

**Fig. 9:**
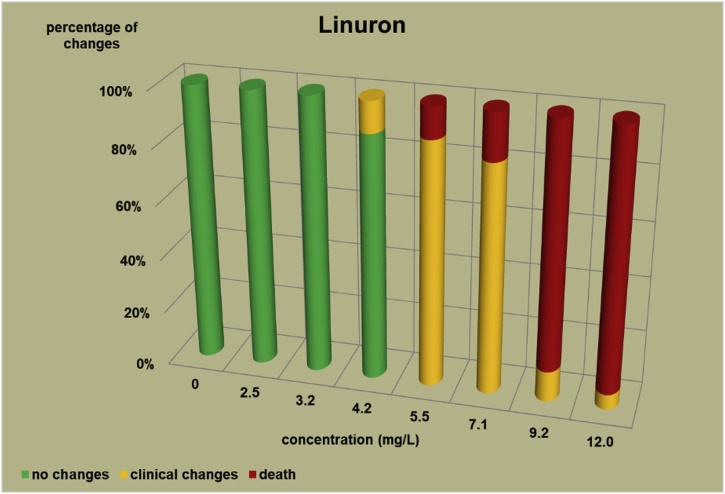
Linuron – changes in zebrafish embryos after 96 h of exposure.

**Fig. 10:**
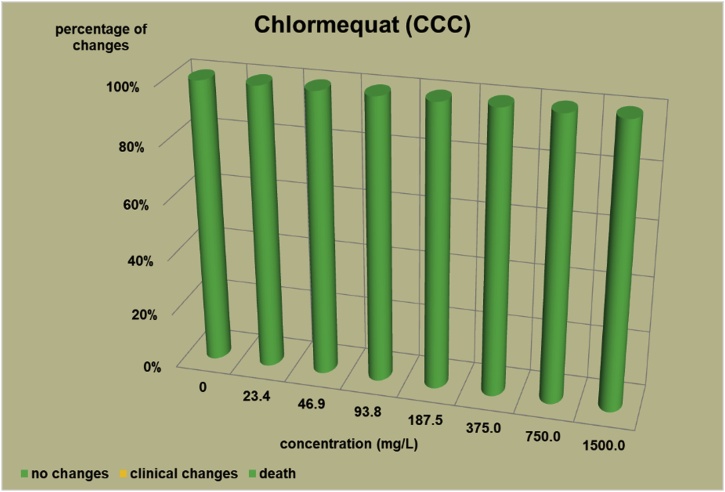
Chlormequat – changes in zebrafish embryos after 96 h of exposure.

**Fig. 11:**
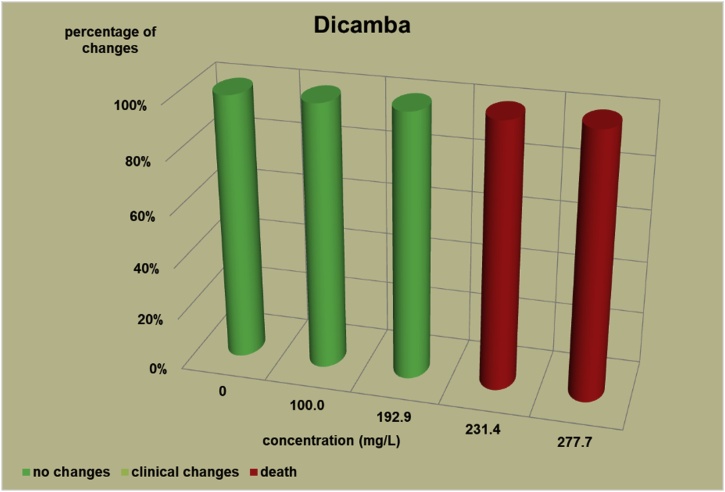
Dicamba – changes in zebrafish embryos after 96 h of exposure.

The changes observed after 96 h, their number and the mortality rate in the zebrafish embryos were presented in the Fig. 1, Fig. 2, Fig. 3, Fig. 4, Fig. 5, Fig. 6, Fig. 7, Fig. 8, Fig. 9, Fig. 10, Fig. 11. The most frequently occurring changes were: edema of the pericardium, edema of the yolk sac and a decrease of the frequency of the heartbeat. These kinds of changes were observed in case of all test items except chlormequat. They seem to be toxic effects, since their intensity was related to the concentration. Less frequent changes were: lordosis, reduction of the body, lateral curvature of the spine, slow development, convulsions when moving, a lack of heartbeat, a heavily deformed body, flexion of the spine. Changes including flexion, curvature, slow development, and deformed body (morphological changes) were observed in case of all test items regarded as embriotoxic, fetotoxic, or teratogenic (2.4-D, carbendazim, MCPA and bromfenvinfos) as well in case of three test items which can have damaging effects on fetuses only at the doses toxic for mothers (glyphosate, prochloraz, deltamethrin).

The Fig. 1, Fig. 2, Fig. 3, Fig. 4, Fig. 5, Fig. 6, Fig. 7, Fig. 8, Fig. 9, Fig. 10, Fig. 11 show the concentrations used in the main tests, after the scope of mortality had been determined. They also present a number of embryos with no changes, or all changes observed in embryos or larvae and dead embryos or larvae after 96 h of exposure. Clinical signs are presented as a sum of all changes observed at a given concentration and time point.

The selection of doses for tests on embryos were chosen to well reflect the relationship between dose and effect, however there were the graphs showing a very steep curve. For carbendazim (Fig. 2) and for glyphosate (Fig. 6) and dicamba (Fig. 11) the factor between concentrations is 1.2 and it is the lowest one used for all tests. The steep curve for glyphosate and dicamba seems to be the specific effect of these substances, despite of low factor between concentrations. For other substances like 2.4-D, MCPA, bromfenvinfos, copper oxychloride, prochloraz, deltametrin and linuron, where the factor between concentrations was higher, the relationship between concentration and effect is much better seen. The factor used in tests for all substances should be the same. It seems that the proper factor between concentrations for these tests is 1.5 which allows to obtain the visible relationship between concentrations and effects for most substances. Proper selection of the same factor between concentrations for all test substances must be carefully considered in future research of this type.

The correlation between the concentration of substances and effects in zebrafish embryos was measured by Pearson correlation coefficient. The coefficient was: for 2.4-D – 0.94; carbendazim – 0.84; MCPA – 0.89; bromfenvinfos – 0.72; copper oxychloride – 0.52; glyphosate – 0.57; prochloraz – 0.68; deltamethrin – 0.57; linuron – 0.84; dicamba – 0.65. The coefficient was high for all compounds with embryotoxic, fetotoxic and teratogenic effects (2.4-D, carbendazim, MCPA and bromfenvinfos) and for linuron. For the rest of the compounds the coefficient was lower.

Changes, which may suggest teratogenic effects (morphological changes), usually occurred in single cases among some tested groups of animals. Therefore, the largest possible group of animals should be investigated in this kind of study [35]. Some changes, which are essential in this kind of study (i.e. flexion, curvature, slow development, deformed body, or lack of some parts of the body), were observed in embryos in the preliminary tests determining mortality. Therefore, the number of embryos exhibiting such changes was included in the total number of embryos, both in the preliminary tests and the main tests. The detailed changes observed in fetuses treated with the test item at used concentrations are presented in the Table 4.

**Table 4:** Changes observed in zebrafish embryos after 96 h of exposure.

Compound	Main tests*		Preliminary tests*	
	Changes in the main tests	Morphological changes in the main tests	Morphological changes in the preliminary tests	
2,4-D	pericardial edema, decrease in the frequency of the heartbeat	lordosis	lordosis	Compound with embryotoxic, fetotoxic and teratogenic effects (strong teratogens)
Carbendazim	pericardial edema, swelling of the yolk sac, decrease in the frequency of the heartbeat	–	flexion of the spine	
MCPA	pericardial edema	–	flexion of the spine	
Bromfenvinfos	pericardial edema, swelling of the yolk sac, decrease in the frequency of the heartbeat	flexion of the spine, shortening the body length, body deformation	flexion of the spine, shortening the body length, body deformation	
Copper oxychloride	pericardial edema, swelling of the yolk sac, decrease in the frequency of the heartbeat	–	–	Toxic effects to fetuses are revealed in maternally toxic doses (maternal toxicity)
Glyphosate	pericardial edema, swelling of the yolk sac, decrease in the frequency of the heartbeat	–	flexion of the spine and flexion of the tail, flexion of the spine, tail short and deformed	
Prochloraz	pericardial edema, swelling of the yolk sac, decrease in the frequency of the heartbeat	flexion of the spine, curvature of tail tip	–	
Deltamethrin	pericardial edema, swelling of the yolk sac, decrease in the frequency of the heartbeat, convulsions	lordosis	–	
Linuron	pericardial edema, swelling of the yolk sac, decrease in the frequency of the heartbeat	–	–	
Chlormequat (CCC)	–	–	–	Lack of fetotoxic and teratogenic effects
Dicamba	pericardial edema, swelling of the yolk sac, decrease in the frequency of the heartbeat	–	–	

* - Main tests - all changes presented, preliminary tests - only morphological changes presented.

### 3.4 Review of the zebrafish embryos tests

In the studies on the zebrafish embryos in which strong teratogens were tested one might notice a joint feature of these test items when the first toxic changes like pericardial edema, swelling of the yolk sac, decrease in the frequency of the heartbeat occur at concentrations considerably lower than the concentrations causing the death of at least half of the embryos and the intensity of changes are greater as the concentrations rises. In the 2,4-D studies (Fig. 1) the first symptoms occurred at a concentration of 62.9 mg/L, while mortality was observed at a concentration of 180.0 mg/l, which was 2.9 as high as the concentration causing symptoms. In the carbendazim studies (Fig. 2) the first symptoms occurred at a concentration of 0.4 mg/L, and considerable mortality was observed at a concentration of 0.7 mg/L, which was 1.8 times as high as the concentration causing symptoms. In the MCPA studies (Fig. 3) the first symptoms occurred at a concentration of 69.28 mg/L, and mortality was observed at a concentration of 200.0 mg/L, which was 2.9 times as high as the concentration causing symptoms. In the bromfenvinfos studies (Fig. 4) the first symptoms occurred at a concentration 0.3125 mg/L, mortality and considerable anatomic changes were observed at a concentration of 2.5 mg/L, which was approximately 8 times as high as the concentration causing symptoms. Another symptom observed in these studies was a flexion of the spine. Some of these changes were observed in the main tests and some of them in the preliminary tests determining the lethal concentrations for embryos, which preceded the main tests (Table 4). The following was observed: in the 2,4-D tests: 1 case of lordosis in the preliminary test and 1 case of lordosis in the main test, in the carbendazim tests: 6 cases of spine flexion in the preliminary tests, in the MCPA tests: 2 cases of spine flexion in the preliminary tests, in the bromfenvinfos tests, apart from 4 cases of spine flexion in the main tests, 3 cases of spine flexion in the preliminary tests. Furthermore, in the bromfenvinfos test, reduction of the body (shortening of the body) and body deformation was observed in 54 larvae in main test as well in 15 embryos in the preliminary tests. Of the other items tested in the embryo studies it was prochloraz and deltamethrin that caused changes similar to those caused by strong teratogen. In case of prochloraz (Fig. 7) the first toxic changes in the embryos were observed at a concentration of 0.98 mg/L, and mortality was observed at a concentration of 5 mg/L, which is approximately 3.3 times as high. What is more, in the prochloraz test 1 case of spine flexion and 1 case of the curvature of a tail tip in the main test were observed. It is important to point out that despite no proven teratogenic effect on fetuses, it causes embryotoxicity (in rat fetuses at a dose of 100 mg/kg) [32]. In case of deltamethrin (Fig. 8) the first toxic changes were observed in fetuses at a concentration of 0.3125 mg/L, inconsiderable mortality was observed at a concentration of 1.25 mg/L, which is approximately 4 times as high as the concentration causing the first symptoms. It is important to point out that in case of deltamethrin numerous cases of lordosis were observed in larvae after hatching (unpublished own research). It might be a sign of strong nerve stimulation causing lordosis. Deltamethrin at higher doses causes convulsions in laboratory animals and humans as well.

In case of the other test items (copper oxychloride, glyphosate, linuron) in which fetus toxicity is connected with maternal toxicity, the changes were weaker than in case of strong teratogens. In the copper oxychloride test (Fig. 5) the first changes were observed at a concentration of 2.5 mg/L, mortality was observed at a concentration of 60 mg/L, which is 24 times as high as the concentration causing the first symptoms. However, in the copper oxychloride test, no deformation of the body was observed (Table 4), while it was caused by strong teratogens. In the glyphosate test (Fig. 6) total mortality was observed at a concentration of 200 mg/L, lower concentrations caused no toxic changes (one case of slight edema of a yolk sac was observed at a concentration of 100 mg/L in preliminary test, which was considered as accidental, no other changes occurred at concentrations ranging from 100 to 200 mg/L). What is more, in the glyphosate studies, in the preliminary tests 3 cases of deformations of the embryo body were observed (Table 4): 1 flexion of the spine and flexion of the tail tip, 1 flexion of the spine and 1 deformation of the tail. In the linuron studies (Fig. 9) toxicity symptoms were observed at all the concentrations causing mortality (5.5, 7.1, 9.2 and 12 mg/L). Some slight changes were observed in 2 embryos at a concentration of 4.2 mg/L (Table 4).

Test items that showed no damaging effects on fetuses (chlormequat and dicamba) caused no toxic changes in live embryos. Chlormequat (CCC) (Fig. 10) proved non-toxic even at a concentration of 1500 mg/L. In the dicamba tests (Fig. 11) the lethal concentrations for the embryos was 231.4 mg/L. However, a concentration at 192.9–1.2 times as low as the lethal concentration did not affect the embryos and larvae within 96 h. In the preliminary tests determining mortality, dicamba proved non-toxic till the 96 h for 9 embryos and larvae at a concentration of 200 mg/L.

It can be concluded that embryo tests cause a relatively straightforward reaction to strong teratogens. However, like in case of the hydra tests, it is difficult to determine clearly the borderline of the toxic effect of strong teratogens on embryos and the effect of test items damaging to fetuses with maternal toxicity. The reaction of the embryos to glifosat, prochloraz or deltamethrin was a similar one to the reaction to strong teratogens such as 2,4-D and bromfenvinfos. It can also be assumed that the mortality at lethal concentration and no toxic changes at the concentrations slightly lower than the lethal ones might suggest no damaging effect on fetuses (the reaction of the embryos to Chlormequat (CCC) and Dicamba). Chlormequat (CCC) proved non-toxic for embryos even at a concentration of 1500 mg/L – a lethal concentration for hydras was 1923 mg/L.

## 4 Conclusions. The scoring system

Both hydra tests and zebrafish embryo tests enabled one to observe clearer damaging toxic effect occurring while exposing hydras and embryos of zebrafish to a strong teratogen, however difficult it is to determine the borderline of no teratogenic effect in the hydra and zebrafish embryo tests using test items that are not strong teratogens, but only show the damaging effect on the development of fetuses with maternal toxicity. To better present the observed changes it seems justifiable to apply a scoring system to evaluate the teratogenic effect on hydras and embryos. The appropriate number of points can be assigned to:

- a given range of the index of the effect on development (TI) values for hydra
- a given range between the concentration which caused mortality and the concentration which caused changes for embryos
- the presence and absence of morphological changes for zebrafish embryos

This proposal is presented in the Table 5.

**Table 5:** Proposed scoring system.

*Classification for hydra*	
index (IT) value	Assigned number of points
From 0 to 1.6	0
From 1.6 to 1.8	1
More than 1.8	2

*Classification for zebrafish embryos*	
The range between the concentration which caused mortality and the concentration which caused changes*	
The range value	Assigned number of points
From 0 to 1.5	0
More than 1.5	1

*The changes observed in the anatomical body structure of the zebrafish embryos (flexion, deformation, slow development, lack of the body parts)*	
Observed changes	Assigned number of points
YES	3
NO	0

An instance of calculating the range of concentration of linuron: the concentration lethal for embryos: 5.5 mg/l, the concentration that is first to cause mortality: 4.2 mg/l, the concentration range: 5,5 : 4,2 mg/l = 1,31, which results in 0 points being given.

* An instance of calculating the range of concentration of 2,4-D: the concentration lethal for embryos: 180 mg/l, the concentration that is first to cause mortality: 62.9 mg/l, the concentration range: 180 : 62,9 mg/l = 2,88, which results in 1 point being given.

The changes observed in the anatomical body structure should be the most important in the suggested scoring system. To evaluate the toxic effect on the zebrafish embryos, an inverted microscope was used. However, far more detailed evaluation methods are possible [30,31], which ensure considerably more detailed evaluation of the skeleton, muscles and nerves like in the evaluation of rats or rabbits.

A more detailed analysis require the use of more advanced techniques like antigens marking, using genetically modified fish, which is far more time consuming. A standard method allows noticing most cases of toxic effect. Morphological changes that this method allows observing are flexion, shrinking of the body or lack of some body parts.

The Table 6 shows how the scoring system results can represent certain parameters, which allows classification of the tests.

**Table 6:** Point evaluation of hydra tests and embryos tests.

	*Hydra: the index of the effect on development (TI)*		*Embryos: range between the lethal concentration and concentration of the first changes*			*Embryos: changes in body structure (flexure, body reduction, body deformation)*			*Sum of the point value*	*Effects on prenatal development in animals or people*
	1	2	3	4	5	6	7	8	9	10
*Test item*	Value of the index (TI) LD50/EC50	Point value	Lethal concentration/concentration with symptoms (mg/l)	Range	Point value	Embryos with change/total number of embryos in both preliminary and main test	Yes/No	Point value		
*2,4-D*	1,396/1,704	1	180,0/62,9	2,9	1	2/153	Yes	3	5	Embryotoxic fetotoxic teratogenic (strong teratogens)
*Carbendazim*	2,04/10,846	2	0,58/0,44	1,5	1	6/153	Yes	3	6	
*MCPA*	1,157/2,169	2	200,0/69,2	2,9	1	2/153	Yes	3	6	
*Bromfenvinfos*	1,242/1,802	2	2,500/0,3125	8	1	76/90	Yes	3	6	
*Copper oxychloride*	1,074/1,285	0	60,0/2,5	24	1	0/108	No	0	1	Toxic effects on fetuses with maternal toxicity
*Glyphosate*	1,243/1,753	1	200,0/200,0	1	0	3/108	Yes	3	4	
*Prochloraz*	1,271/1,316	0	5,0 / 1,5	3,3	1	2/108	Yes	3	4	
*Deltamethrin*	5,091/1,967	2	1,2500/0,3125	4	1	48/72	Yes	3 (0)*	6 (3)*	
*Linuron*	1,261/1,144	0	5,5/4,2	1,3	0	0/144	No	0	0	
*Chlormequat (CCC)*	1,142/1,311	0	No/No	0	0	0/225	No	0	0	No negative effects on the fetuses
*Dicamba*	1,129/1,649	1	231,4/231,4	1	0	0/144	No	0	1	

* Assuming that lordosis is the result of stimulation of the nervous system, the final result for deltamethrin is 3.

The column No. 2 presents the point values (0, 1 or 2) assigned to the size of the LD50 or EC50 index of the effect on development (TI) of each test item presented in the column No. 1.

The column No. 5 presents the point values (0 or 1) assigned to the range between the lethal concentration and the concentration of the first changes of each test item presented in the column No. 4.

The column No. 8 presents the point values (0 or 3) assigned to lack of morphological changes or occurrence of morphological changes in embryos presented in the column No. 7.

Point values in columns 2, 5 and 8 are assigned according to the Table 5. The numbers in the column No. 9 are the sum of columns 2, 5 and 8, and they are the final result of the classification of each test item. The column No. 10 classifies all test items into three groups depending on the effect on the animal or human fetuses.

In this system, strong teratogens are assigned 5 or 6. If a test item was given fewer than 5 points, that means it had no toxic teratogenic effect without determining whether it is or not an item having damaging effect on the development with maternal toxicity.

Hydra attenuata is an undemanding creature, easy to breed. Simple conditions and unrefined breeding methods make it possible to obtain a large number of individuals for testing. It is assumed that the regeneration process to some extent reflects fetal development, however, it should be remembered that this is a very simplified model, far from the actual processes that take place in the fetal life of vertebrates. Calculation of the index (TI) value is based on the direct toxicity for adults and disturbances in the regeneration process of the gastric parts of the hydra, therefore it should be emphasized that the toxicity of the tested material for adults strongly influences the final value of index (TI). Taking these limitations into account, tests on that animal should be considered as auxiliary and additional.

Danio rerio is more difficult species to breed. They need much better and stable conditions, and it is more difficult to obtain the proper amount of good quality embryos. The processes that take place during the development of the embryo much better reflect the antenatal development of mammals, therefore the changes that we can observe in tests on embryos must be of greatest importance. This is confirmed by the results that all potent teratogens showed a strong response in embryos.

Studies of the usefulness of zebrafish embryotoxicity tests to assess prenatal toxicity using are being carried out, on both zebrafish embryos alone [36] and in combination with other alternative tests [37]. The data of these tests often show that the results observed in the tested models are consistent with the results of full tests in laboratory animals in vivo [38]. These relatively simple tests on hydra and danio embryos with proposed scoring system presented in this paper also confirms that the results observed are consistent with those obtained on laboratory animals. Therefore, quite reasonable way is the more and more widespread use of danio embryos alone [39] as well as systems (batteries) consisting of alternative tests and danio embryos [37].

Despite the large number of in vitro and alternative methods developed, these methods cannot currently replace animal testing in themselves. They can certainly be an auxiliary element constituting a cross-cutting approach limiting the use of animals [40]. The study combining hydra and zebrafish tests seems to have the potential of an auxiliary test, although the test's effectiveness and reliability should be tested on more compounds.

## Author contributions

**Robert Sornat:** Funding acquisition, Methodology, Project administration, Resources, Formal analysis, Supervision, Investigation, Data curation, Writing - original draft.

**Joanna Kalka:** Formal analysis, Supervision.

**Justyna Faron:** Methodology, Data curation, Investigation, Formal analysis, Writing - review & editing.

**Marta Napora-Rutkowska:** Methodology.

**Daniel Krakowian:** Methodology, Software, Data curation, Formal analysis, Writing - review & editing.

**Agnieszka Drzewiecka:** Methodology, Data curation.

## Ethical approval

All applicable international, national, and/or institutional guidelines for the care and use of animals were followed.

According to European and Polish laws, the use of the animals described in this article did not require the approval of the Ethical Committee.

This article does not contain any studies with human participants performed by any of the authors.

The authors agree with the publication of the manuscript in this form.

## Funding

This study was funded by Ministry of Science and Higher Education (contract number: 4180/E-142/S/2014 of 19.02.2015).

## Declaration of Competing Interest

The authors declare that they have no known competing financial interests or personal relationships that could have appeared to influence the work reported in this paper.
